# Experimental DNA Demethylation Associates with Changes in Growth and Gene Expression of Oak Tree Seedlings

**DOI:** 10.1534/g3.119.400770

**Published:** 2020-01-15

**Authors:** Luke Browne, Alayna Mead, Courtney Horn, Kevin Chang, Zeynep A. Celikkol, Claudia L. Henriquez, Feiyang Ma, Eric Beraut, Rachel S. Meyer, Victoria L. Sork

**Affiliations:** * Department of Ecology and Evolutionary Biology,; † La Kretz Center for California Conservation Science, Institute of the Environment and Sustainability,; ‡ Molecular Biology Institute,; § Department of Molecular, Cellular, and Developmental Biology, and; ** Institute of the Environment and Sustainability, University of California, Los Angeles, CA, 90095

**Keywords:** DNA methylation, *Quercus lobata*, 5-Azacytidine, leaf morphology, gene expression

## Abstract

Epigenetic modifications such as DNA methylation, where methyl groups are added to cytosine base pairs, have the potential to impact phenotypic variation and gene expression, and could influence plant response to changing environments. One way to test this impact is through the application of chemical demethylation agents, such as 5-Azacytidine, which inhibit DNA methylation and lead to a partial reduction in DNA methylation across the genome. In this study, we treated 5-month-old seedlings of the tree, *Quercus lobata*, with foliar application of 5-Azacytidine to test whether a reduction in genome-wide methylation would cause differential gene expression and change phenotypic development. First, we demonstrate that demethylation treatment led to 3–6% absolute reductions and 6.7–43.2% relative reductions in genome-wide methylation across CG, CHG, and CHH sequence contexts, with CHH showing the strongest relative reduction. Seedlings treated with 5-Azacytidine showed a substantial reduction in new growth, which was less than half that of control seedlings. We tested whether this result could be due to impact of the treatment on the soil microbiome and found minimal differences in the soil microbiome between two groups, although with limited sample size. We found no significant differences in leaf fluctuating asymmetry (*i.e.*, deviations from bilateral symmetry), which has been found in other studies. Nonetheless, treated seedlings showed differential expression of a total of 23 genes. Overall, this study provides initial evidence that DNA methylation is involved in gene expression and phenotypic variation in seedlings and suggests that removal of DNA methylation affects plant development.

Epigenetic processes, such as DNA methylation or histone modifications, have been well studied for their role in gene expression, cell development, and transposon silencing (Law and Jacobsen 2010; Lisch 2009; Schmitz *et al.* 2011; Dowen *et al.* 2012). Recently, epigenetic modifications have garnered interest from ecologists and evolutionary biologists for their potential role in plant response to the environment (Bossdorf *et al.* 2008; Richards *et al.* 2017; Robertson and Wolf 2012; Verhoeven *et al.* 2016). Epigenetic modifications are a potential mechanism for long-lived species like trees to cope with rapidly changing environmental conditions within their lifespan (Balao *et al.* 2018; Bräutigam *et al.* 2013). Indeed, variation in DNA methylation, one of the most well-studied epigenetic modifications, has been shown to be associated with ecologically and evolutionarily important phenotypic traits like flowering time and growth in short-lived species (Bossdorf *et al.* 2010; Cortijo *et al.* 2014; Finnegan *et al.* 1996; Herman and Sultan 2016; Johannes *et al.* 2009; Zhang *et al.* 2013), although much less is known for tree species (but see Bräutigam *et al.* 2013). The few studies that have focused on trees have found associations between DNA methylation and climate gradients in natural populations of oaks (*Quercus lobata*) (Gugger *et al.* 2016), between temperature and embryogenesis, possibly through epigenetic regulation in Norway Spruce (*Picea abies*) (Yakovlev *et al.* 2011), and epigenetic responses of poplar (*Populus trichocarpa*) to drought stress (Liang *et al.* 2014). These studies suggest that epigenetic variation affects phenotype, but we still lack an understanding of the associations between epigenetic variation, gene expression, and phenotypic traits in trees (Bräutigam *et al.* 2013), which are among the most ecologically and economically important plant taxa (Brockerhoff *et al.* 2017).

The overall role of DNA methylation in gene regulation and phenotypic variation remains enigmatic (Niederhuth and Schmitz 2017). Among the most well-studied epigenetic modifications is cytosine DNA methylation (Richards *et al.* 2010; Verhoeven *et al.* 2010; Verhoeven *et al.* 2016), where cytosine bases are methylated in CG, CHG, or CHH sequence contexts (H = A, T, or C) through distinct molecular pathways (Finnegan *et al.* 1996; Law and Jacobsen 2010). Importantly, the effects of DNA methylation on gene expression and phenotypic traits depend on both the sequence context and genomic location (*e.g.*, promoter regions or gene bodies, Niederhuth and Schmitz 2017). For example, methylation in the promoter regions of genes in the CHH context is commonly associated with repressed transcription (Liang *et al.* 2014; Secco *et al.* 2015; Vining *et al.* 2012; Zhang *et al.* 2006), and may be associated with silencing of transposable elements. In contrast, CG gene body methylation is most often associated with high transcriptional activity (Dubin *et al.* 2015; Niederhuth and Schmitz 2017; Zilberman *et al.* 2007). However, the loss of CG gene body methylation in *Arabidopsis thaliana* epigenetic recombinant inbred lines does not lead to differences in gene expression (Bewick *et al.* 2016), and studies in poplar (*Populus sp*.) have found both higher (Liang *et al.* 2014) and lower (Vining *et al.* 2012) gene expression levels associated with CG gene body methylation. These findings highlight a critical need to understand how DNA methylation in different sequence contexts, gene expression, and phenotype are linked to elucidate the overall function of DNA methylation in the plant genome and its potential role in plant response to environmental change.

One approach to elucidating the associations between DNA methylation, phenotype, and gene expression is through experimentally demethylating individuals using chemicals that interfere with the methylation process, such as 5-Azacytidine. 5-Azacytidine acts as a non-methylable cytosine analog, incorporating itself into the genome during DNA replication and leading to non-targeted partial demethylation across the genome in all sequence contexts (Chang and Pikaard 2005; Cheng *et al.* 2004; Griffin *et al.* 2016; Pecinka and Liu 2014). In addition, 5-Azacytidine acts to inhibit the action of DNA methyltransferases in the cell (Cheng *et al.* 2004; Christman *et al.* 1983; Creusot *et al.* 1982; Jones 1985), contributing to partial genome-wide demethylation. A reduction in DNA methylation, associated with the application of 5-Azacytidine or other experimental methods, has led to phenotypic changes such as decreased growth, higher mortality, changes in leaf morphology, and altered flowering time (Bossdorf *et al.* 2010; Burn *et al.* 1993; Fieldes and Amyot 2000; Finnegan *et al.* 1996; Vergeer *et al.* 2012). 5-Azacytidine is commonly applied as a solution during seed germination, though reduced vigor and growth is a common side-effect of this treatment (Akimoto *et al.* 2007; Amoah *et al.* 2012; Bossdorf *et al.* 2010), potentially due to limited root development (Kanchanaketu and Hongtrakul 2015; Puy *et al.* 2017). Recently, Puy *et al.* (2017) demonstrated that the foliar application of 5-Azacytidine may minimize these unwanted side effects and provided a method to experimentally demethylate established seedlings after germination has occurred. Additionally, this method is feasible for plants that are not easily germinated on filter paper or in petri dishes (*e.g.*, tree species with large seeds), which expands the opportunities to test the effects of experimental demethylation on phenotypic variation and gene expression in a wider range of plant species. To our knowledge, the foliar application of 5-Azacytidine has not been tested in a woody plant species, nor in individuals older than a few months.

In this study, we tested whether the foliar application of 5-Azacytidine to *Quercus lobata* (valley oak) seedlings is associated with changes in phenotypic variation and gene expression. Valley oak is an ecologically and culturally important woody species endemic to California (Pavlik *et al.* 1991). Variation in DNA methylation across populations of valley oak show signatures of association with adaptation to the environment (Gugger *et al.* 2016; Platt *et al.* 2015). Yet, the question remains whether trends in DNA methylation have any direct association with phenotypic variation and gene expression in this species, or more broadly in other hardwood species. If there is a relationship, we would expect that the foliar application of 5-Azacytidine would lead to an overall reduction in genome-wide DNA methylation across all sequence contexts similar to previous studies (Griffin *et al.* 2016; Puy *et al.* 2017). Furthermore, we would expect that if DNA methylation is mechanistically linked to phenotype and gene expression, then experimental reduction in DNA methylation would lead to phenotypic differences in seedlings, specifically in the amount of total new growth and degree of leaf fluctuating asymmetry, and differential gene expression across treatment and control samples. Because 5-Azacytidine may affect the development and survival of bacteria and fungi (Čihák 1974; Lal *et al.* 1988; Lin *et al.* 2013; Tamame *et al.* 1983), we also compared the soil microbiome of samples treated with 5-Azacytidine *vs.* control samples to assess whether phenotypic or gene expression differences related to the treatment could be explained by indirect effects of 5-Azacytidine on the soil microbiome community.

## Materials and Methods

### Study species

**Figure 1:**
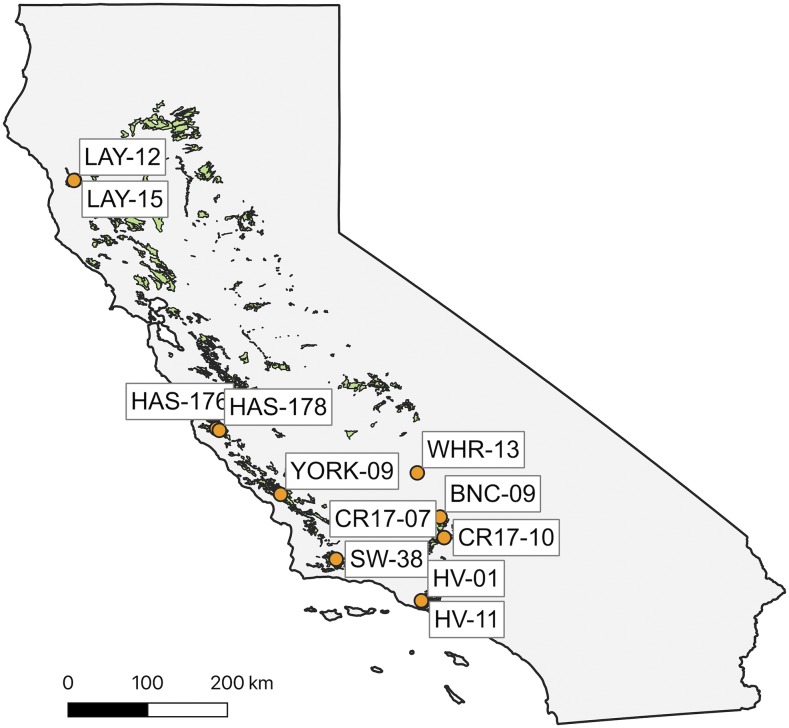
Map of California showing locations of sampled *Quercus lobata* (valley oak) populations (orange circles) with corresponding labels. Contemporary species range of *Quercus lobata* is shaded in green.

*Quercus lobata Neé* (valley oak) is a foundational oak species endemic to California woodlands and savannas with high cultural and ecological importance (Anderson 2007; Pavlik *et al.* 1991). The species ranges from 0-1,700 m in elevation (Griffin and Critchfield 1972), with populations spanning a large latitudinal gradient across California (Figure 1). Because of habitat loss and conversion, the current range of valley oak is highly fragmented, with many populations experiencing sharp declines over the past century (Whipple *et al.* 2011) and are now further threatened by climate change (Kueppers *et al.* 2005; Sork *et al.* 2010). Previous studies on DNA methylation in *Q. lobata* have found significant differentiation across populations, particularly in the CG context (Platt *et al.* 2015) as well as strong associations between DNA methylation and climate variation, with temperature and CG methylation showing the strongest associations (Gugger *et al.* 2016).

### Acorn collection and planting

From October-November 2017, we collected open-pollinated acorns of *Quercus lobata* from 8 localities across California: Bakersfield National Cemetery (BNC), Cordon Ridge (CR), Hastings Reserve (HAS), Hidden Valley (HV), Laytonville (LAY), Sedgwick Reserve (SW), White River (WR), York Mountain Road (YORK) (Figure 1, Table S1). We collected a total of 12 families (each corresponding to an individual maternal tree) across the 8 localities, with 2 families in each of Laytonville, Hastings Reserve, and Cordon Ridge, and one tree each from the remaining localities. Because valley oak has extensive wind-pollination, we assume each family of acorns consists of primarily half-siblings, with occasional full siblings possible (Grivet *et al.* 2009). We planted the acorns in the UCLA Plant Growth Center on December 20, 2017 in Stuewe & Sons D40 6.9 cm × 25.4 cm Deepot containers using homogenized and autoclaved soil. Seedlings were watered as needed and periodically sprayed with fungicide to control powdery mildew.

### Application of 5-Azacytidine

We began the experiment on May 18, 2018, when seedlings were approximately 5 months old. Because we expected to observe the greatest effect of demethylation on young developing tissue and to ensure that our treatment and control samples were at a similar developmental stage, we pruned all seedlings in the experiment to 10 cm in height. Valley oak seedlings readily re-sprout when pruned, which allowed us to test the effect of experimental demethylation on emerging leaves. We designated three randomly chosen seedlings from each family to receive either the 5-Azacytidine treatment or a control spray, for a total of 72 seedlings in the experiment (n = 36 treatment, n = 36 control). We placed seedlings in a growth chamber at 25° on a 12-hour light/dark cycle and watered as needed, separating the treatment and control samples to minimize cross-contamination. Seedlings were sprayed for a total of 27 days (May 18, 2018 to June 13, 2018). The 5-Azacytidine treatment consisted of daily spraying of a solution of 50 µM 5-Azacytidine in 1.5% TWEEN20 (*i.e.*, a surfactant used to help increase the efficiency of 5-Azacytidine) (Puy *et al.* 2017). The control seedlings were sprayed on the same schedule with a solution of 1.5% TWEEN20. Prior to the experiment we tested for toxicity across different concentrations of TWEEN20 (0.5%, 1.0%, and 1.5%) on a separate set of seedlings and observed no visible differences in leaves after one week for any of the three concentrations. However, during the experiment, after two weeks, signs of surfactant induced leaf damage was observed in both treated and control samples and the concentration of TWEEN20 was reduced to 1% for the remainder of the experiment. We concluded the experiment on June 14, 2018 when we measured phenotypes of the treated and control seedlings and collected tissue for RNA-seq and whole-genome bisulfite sequencing. We flash froze collected tissue in liquid nitrogen, then transported the tissue to the lab on dry ice and stored in the -80° freezer until the day of extractions.

### Phenotypic measurements

At the end of the experiment (June 14, 2018), we measured the total amount of new vegetative growth and fluctuating asymmetry of leaves from treated and control seedlings. We defined total new growth as the summed length in centimeters of all newly developed shoots following pruning at the start of the experiment. Because all seedlings were pruned to 10 cm prior to the start of the experiment, new shoots were easily identified. We obtained phenotypic measurements of growth of 63 seedlings (n = 32 treatment, n = 31 control, Table S1). We used an ANOVA to test for differences of total new growth between treated and control samples, while including family as a fixed effect in R 3.5.1 (R Core Team 2018).

To measure leaf fluctuating asymmetry (*i.e.*, deviations from bilateral symmetry), we collected on average 3.3 leaves from 67 individuals (range: 1 – 6 leaves per individual, 219 leaves total, Table S1). We cut each leaf along the midrib and measured the leaf area of each leaf half with the image processing program ImageJ (Rueden *et al.* 2017) after scanning on a flat-bed scanner. Following Palmer and Strobeck (1986), we measured fluctuating asymmetry of leaves using two indices. The first index (FA_1_) for an individual *i* measures the absolute value of the difference in area between the right (R_i_) and left (L_i_) half of each leaf, average across *N* leaf measurements:

$$FA_{1}=\;\frac{\sum^{\text{​}}|R_{i}-\;L_{i}|}{N}$$

The second index is similar, though it scales the index by leaf size:

$$FA_{2}=\;\frac{\sum^{\text{​}}\frac{\left|R_{i}-\;L_{i}\right|}{(R_{i}+\;L_{i})/2}}{N}$$

We used an ANOVA to test for differences in leaf fluctuating asymmetry between treated and control samples, while including family as a fixed effect in R 3.5.1 (R Core Team 2018).

### Whole Genome Bisulfite Sequencing (WGBS)

To ensure that our plants were demethylated and to quantify the effect of 5-Azacytidine treatment on genome-wide methylation levels, we performed low coverage Whole Genome Bisulfite Sequencing on a subset of four seedlings – two half-siblings, one control and one treated, from two families (HV-11-5, HV-11-33, and LAY-15-30, LAY-15-34, n = 2 treatment, n = 2 control, Table S1).We extracted total genomic DNA from frozen leaf tissue from these seedlings on June 18, 2018 using a prewash method (Li *et al.* 2007) followed by a modified CTAB protocol (Doyle and Doyle 1987). The four seedlings were chosen based on the most notable phenotypic differences between treatment and control seedlings in each family (Figure S1). Plants were frozen in liquid nitrogen and ground using a Mixer Mill MM301 (Retsch, Germany). The prewash method was repeated up to 3x until a clear supernatant was achieved. The resultant pellet was then used in a modified CTAB protocol in which the chloroform-isoamyl (24:1) step was repeated twice. DNA was quantified using the Qubit dsDNA BR Assay Kit on the Qubit 3.0 Fluorometer (Life Technologies, Carlsbad, CA).

Total genomic DNA at a concentration of 500 ng in 60uL was sonicated using an S2 Focused-ultrasonicator (Covaris, Woburn, MA, USA) for 60 sec to obtain fragments in the 200-300 bp range (Duty cycle: 10%, intensity: 5, cycles/burst: 200, Mode: Frequency sweeping). Kapa DNA hyper kit (Kapa Biosystems, Inc., MA, USA) was used to repair the ends of the DNA fragments and to ligate the DNA fragments with Illumina TruSeq DNA LT Nano adapters. Subsequently, the adapter ligated DNA was treated with the Epitect bisulfite kit (Qiagen, Hilden, Germany). Finally, bisulfite treated DNA was PCR amplified once with MyTaq DNA polymerase (Bioline, MA, USA) at 15 cycles. WGBS libraries were pooled and sequenced on an Illumina HiSeq 4000 machine. The reads were aligned to v3.0 of the *Quercus lobata* genome (Sork *et al.* 2016 (https://valleyoak.ucla.edu/genomic-resources/)) using BSseeker2 (Guo *et al.* 2013) and bowtie with default parameters (Table S2). After alignment, BSseeker2 was used again to call methylation with default parameters. CgmapTools (Guo *et al.* 2018) were then used to analyze the methylation levels for each sample.

### RNAseq Library preparation and sequencing

We performed RNA sequencing on leaf tissue in a subset of seedlings to test whether the demethylation treatment altered gene expression. Total RNA was extracted from the frozen leaf tissue of two control and two treated half-siblings from each of three families (HV-11, LAY-15, and YORK-09, Table S1), which were selected due to the large phenotypic differences observed between control and treated plants. In total, we sequenced RNA from 12 seedlings (n = 6 treatment, n = 6 control). Three separate RNA extractions were performed on different days between October 16-30, 2018 using a modified version of the Conifer RNA prep protocol from the Cronn Lab and the Spectrum Plant Total RNA kit (Sigma, St. Louis, MO, USA). Leaf tissues were flash frozen in liquid nitrogen and ground using a Mixer Mill MM301 (Retsch, Germany). Powdered tissues were transferred to cold 2mL tubes and 1.8 mL of cold RNA Extraction Buffer + DTT was added. RNA Extraction Buffer consists of 8 M Urea, 3 M LiCl, 1% polyvinylpyrrolidone K-60, 5 mM DTT (added just before use, 1M stock). The tubes were then vortexed for 30 sec, incubated at 4° for 30 min, then centrifuged at 4° for 30 min at 20,000 r*cf*. The supernatant was discarded and the pellet was used as the starting material for the Spectrum Plant Total RNA kit, protocol A, adding 750uL of Binding Solution, and performing on-column DNase I digestion. RNA quality and quantity were assessed using the Agilent RNA ScreenTape System on the Agilent 2200 TapeStation system (Agilent Technologies, Santa Clara, CA, USA).

RNAseq libraries were constructed on November 14, 2018 using the TruSeq RNA Library Prep Kit v2 (Illumina, San Diego, CA, USA) following the Low Sample (LS) Protocol in the TruSeq RNA Sample Preparation v2 Guide. Briefly, mRNA was purified and fragmented from total RNA at a concentration of 1 µg in 50 µL using poly-T oligo-attached magnetic beads. First strand and second strand cDNA synthesis, end repair, dA-tailing, ligation, purification, and enrichment steps were performed following the manufacturer’s instructions. Libraries were analyzed using the Agilent D1000 Screen Tape System on the Agilent 2200 TapeStation (Agilent Technologies, Santa Clara, CA, USA). Libraries were quantified using the Qubit dsDNA BR Assay Kit on the Qubit 3.0 Fluorometer (Life Technologies, Carlsbad, CA). Libraries were pooled and sequenced on the Illumina HiSeq 4000 sequencer using paired-end 100 bp reads.

### RNAseq data processing

Raw sequenced reads were converted from qseq to fastq format, reads failing the Illumina quality filter were removed, and reads were demultiplexed using custom scripts (available at github.com/alaynamead/RNAseq_scripts). Sample quality was checked using FastQC 0.11.8 (Andrews 2010). Cutadapt 2.3 (Martin 2011) was used to trim adapters and low quality ends (using a cutoff quality score of 27) and reads shorter than 30 bp after trimming were removed. Reads were aligned to v3.0 of the *Quercus lobata* genome using STAR 2.7.1 (Dobin *et al.* 2013). Reads which mapped to multiple locations within the genome (quality score < 255) were removed using Samtools 1.9 (Li *et al.* 2009). The Picard 2.20.3 MarkDuplicates function (Broad Institute 2019) was used to identify duplicate reads, and the sequencing-platform artifact duplicates were removed. The number of reads mapping to each gene was obtained using the HTSeq 0.11.1 htseq-count function (Anders *et al.* 2015).

### Differential expression analysis

Genes that were differentially expressed between demethylated and control seedlings were identified using the R package DESeq2 (Love *et al.* 2014). Before analysis, lowly expressed genes were removed using the filterByExpr function from the edgeR package (McCarthy *et al.* 2012) to keep only genes having a count-per-million above 15 across all samples and above 10 in at least two samples (in order to avoid removing genes that were expressed in only one treatment/family group). After filtering, 23,487 genes remained and were used for differential expression analysis. We identified genes that were differentially expressed in seedlings treated with 5-Azacytidine *vs.* control using the model “expression ∼ treatment + RNA extraction date” in order to control for potential batch effects from three sets of RNA extractions. We also tested whether treatment altered gene expression for each family separately.

We adjusted P-values using the Benjamini-Hochberg correction and considered genes with an adjusted p-value < 0.05 to be differentially expressed. Raw gene counts were normalized for visualization using DESeq2’s varianceStabilizingTransformation function, which transforms counts to continuous values and normalizes gene expression based on library size and gene variance. PCAs on the transformed gene expression values were used to visualize sample clustering using R’s prcomp function. The R package GOseq (Young *et al.* 2010) was used to identify gene ontology terms that were enriched in the upregulated or downregulated genes for each model tested.

### Soil microbiome sampling

At the end of the experiment, we collected a 2mL sample of topsoil from a total of 12 seedlings (n = 6 treatment, n = 6 control, Table S1). The soil was vortexed to mix it and 0.25g of soil was used for DNA extraction with the DNEasy PowerSoil Kit. DNA was used in multilocus metabarcoding for two loci: 16S targeting bacteria and archaea and Fungal ITS targeting fungi. Primer sequences were as follows. 16S 515f: GTGYCAGCMGCCGCGGTAA, 806r: GGACTACNVGGGTWTCTAAT (Caporaso *et al.* 2012). Fungal ITS 5F: GGAAGTAAAAGTCGTAACAAGG, 5.8SR: CAAGAGATCCGTTGTTGAAAGTT (Epp *et al.* 2012). This first PCR was performed using a 15uL reaction mixture containing 7.5 uL QIAGEN Multiplex Plus Taq PCR 2x Master Mix, 3nM of each primer, and 2uL of DNA template. The first PCR was done in triplicate with the above primers containing Illumina (San Diego, CA) Nextera adaptor sequences in the 5′ primer sequence, then products were confirmed by gel electrophoresis, cleaned using Sera-Mag Serapure beads (Sigma-Aldrich, Darmstadt, Germany), quantified using the Qubit DNA BR Assay (Thermo Fisher, Walthamm, MA) and pooled at equimolar levels by sample. Illumina Nextera indices were added through an additional indexing PCR done with Kapa HiFi HotStart Ready Mix (Roche, Indianapolis, IN, USA) according to manufacturer’s guidelines. Indexed samples were confirmed by gel electrophoresis, cleaned, quantified, and pooled as described in the first PCR, except samples were pooled together rather than primers.

DNA libraries were sequenced on an Illumina MiSeq with reagent kit S3 for 600 cycle (2 × 300bp reads) aiming for 25,000 reads in each direction per locus. Fastq libraries were analyzed using the default settings of the Anacapa Toolkit (Curd *et al.* 2019) and filtered conservatively to remove any taxa in DNA or PCR controls at >2 reads. Conservative filtering was used rather than model-based filtering because the small sample size would have low power in models. For the ITS results, only two taxa were removed, both from the genus *Ramularia* that were present in only two total reads across the 12 samples. For the 16S results, 21 taxa were removed, 10 of which were only present in the negative control, and 8 of which were ubiquitously present in samples.

The decontaminated results, as reads per sample per taxon lists and sample metadata of parent provenance, treatment, and planter, were converted to PhyloSeq objects using ranacapa (Kandlikar *et al.* 2018), analyzed using PhyloSeq (McMurdie and Holmes 2013) and vegan (Oksanen *et al.* 2015) in R. Samples were rarified to even depth using the PhyloSeq function “rarefy_even_depth” that removed 150 taxa leaving 1070 taxa in the 16S results and that removed 50 taxa leaving 220 taxa in the ITS results set. Alpha Diversity was calculated using observed richness, Shannon, Simpson, and Chao1 indices. Analysis of Similarity ANOSIM analysis with 999 free permutations was used to examine whether there were significant differences community composition using the Bray-Curtis, Jaccard, and Chao estimators across treatment *vs.* control groups, by planter, and locality.

### Data availability

Raw bisulfite sequencing reads are available in the NCBI SRA under accession number PRJNA575572. DNA methylation levels by sample and chromosome and phenotypic data on total new growth and leaf fluctuating asymmetry are publicly available through a figshare repository (DOI: 10.6084/m9.figshare.9932360). Raw reads and expression levels by gene from the RNA-seq analysis are available on NCBI GEO under accession number GSE138108. Scripts for processing RNA-seq data are available at http://github.com/alaynamead/RNAseq_scripts. Raw sequences from the soil microbiome are available in the NCBI SRA under accession number PRJNA575572 and the complete OTU list is available on a figshare repository (https://doi.org/10.6084/m9.figshare.9932360).

## Results

### Effects of 5-Azacytidine on genome-wide methylation levels

**Figure 2:**
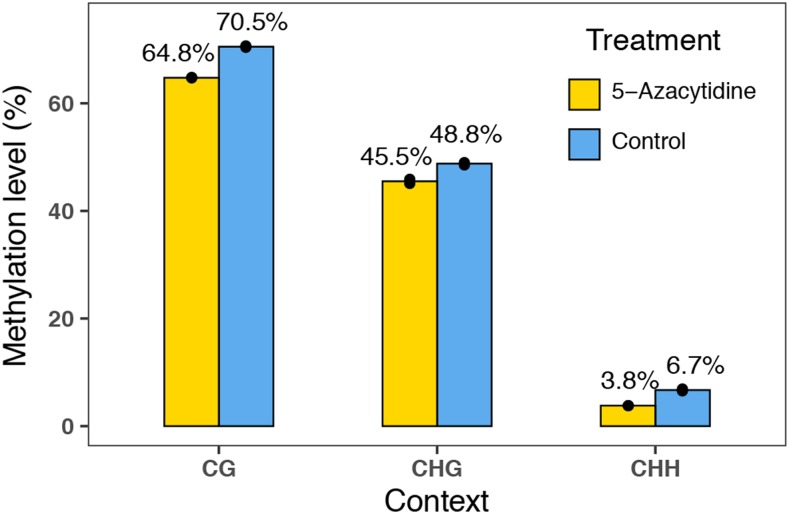
Effect of 5-Azacytidine treatment on genome-wide methylation levels (%) across the three sequence contexts found in two seedlings of *Quercus lobata* per experimental group. Yellow indicates samples treated with the demethylating agent 5-Azacytidine, and blue indicates control samples. Black circles indicate data points for individual samples (n = 2 for each treatment).

We grew a total of 72 *Q. lobata* wild-collected seedlings from 12 families (Figure 1) in a greenhouse and when the seedlings were approximately 5 months old, we performed daily foliar application of 5-Azacytidine on half the seedings (n = 36 treatment, n = 36 control) for 27 days. At the end of the experiment, we ran low coverage (∼1x) whole genome bisulfite sequencing on the leaf tissue of four seedlings that showed notable phenotypic differences (n = 2 treatment, n = 2 control, Figure S1) and observed a 3–6% reduction in genome-wide methylation levels in leaf tissue of seedlings treated with 5-Azacytidine compared to controls (Figure 2). Methylation in the CG context showed the highest absolute reduction in methylation levels of 6%, while CHG and CHH both showed reductions of 3%. In terms of the relative reductions between treated and control seedlings, CG methylation decreased by 8.2%, CHG by 6.7%, and CHH by 43.1%. The pattern of reduced methylation following treatment with 5-Azacytidine was consistent across the 12 valley oak chromosomes (Figure S2). Read coverage of cytosines across the genome was similar across samples (range: 1.080-1.096x).

### Effects of 5-Azacytidine on phenotypic variation

**Figure 3:**
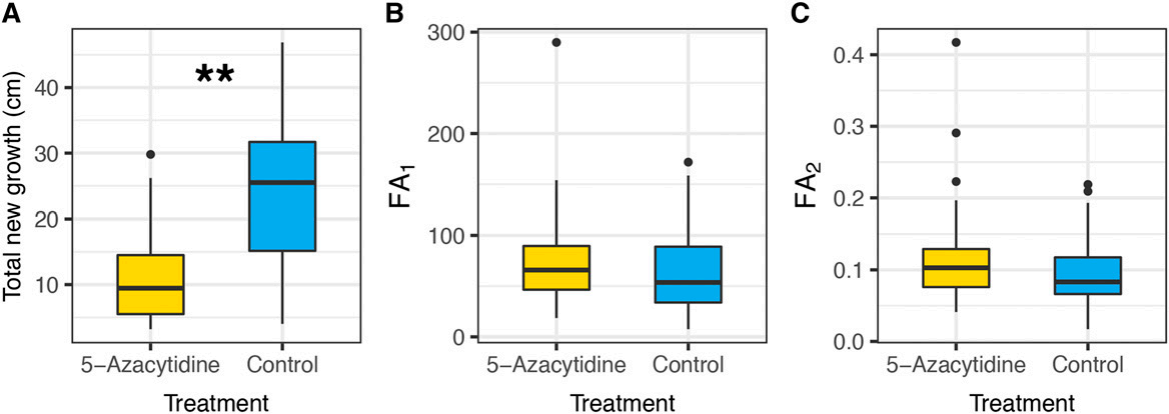
Phenotypic measurements of *Quercus lobata* seedlings for (a) total new growth (F = 41.77, df = 1, *P* = < 0.001), (b) leaf fluctuating asymmetry not scaled by leaf size (FA_1_, F = 1.17, df = 1, *P* = 0.285), and (c) leaf fluctuating asymmetry scaled by leaf size (FA_2_, F = 1.55, df = 1, *P* = 0.219). Total new growth is lower in valley oak seedlings treated with demethylating agent 5-Azacytidine compared to control seedlings (indicated by **), while there were no statistically significant differences in either index of fluctuating asymmetry.

At the end of the experiment, we measured the total amount of new growth and leaf morphology on treated and control seedlings. Seedlings treated with 5-Azacytidine showed lower amounts of total new growth compared to control seedlings (Figure 3a, F = 41.77, df = 1, *P* = < 0.001). Treated seedlings grew on average 10.9 ± 6.6 cm (mean ± SD) compared to control seedlings 24.1 ± 10.2 cm. Overall total new growth also differed among families (F = 2.68, df = 11, *P* = 0.0087), though the overall pattern of lower growth for treated samples was consistent across 11/12 families (Figure S3).

Seedlings treated with 5-Azacytidine did not show statistically significant differences in leaf fluctuating asymmetry (Figure 3b FA_1_: F = 1.17, df = 1, *P* = 0.285; Figure 3c FA_2_: F = 1.55, df = 1, *P* = 0.219). Fluctuating asymmetry showed statistically significant differences among families for the first index (FA_1_) that does not scale by leaf size (Figure S4, F = 2.03, df = 11, *P* = 0.043) but not the second index of fluctuating asymmetry (FA_2_) that scales by leaf size (Figure S4, F = 1.60, df = 11, *P* = 0.124).

### Effects of 5-Azacytidine on gene expression

**Figure 4:**
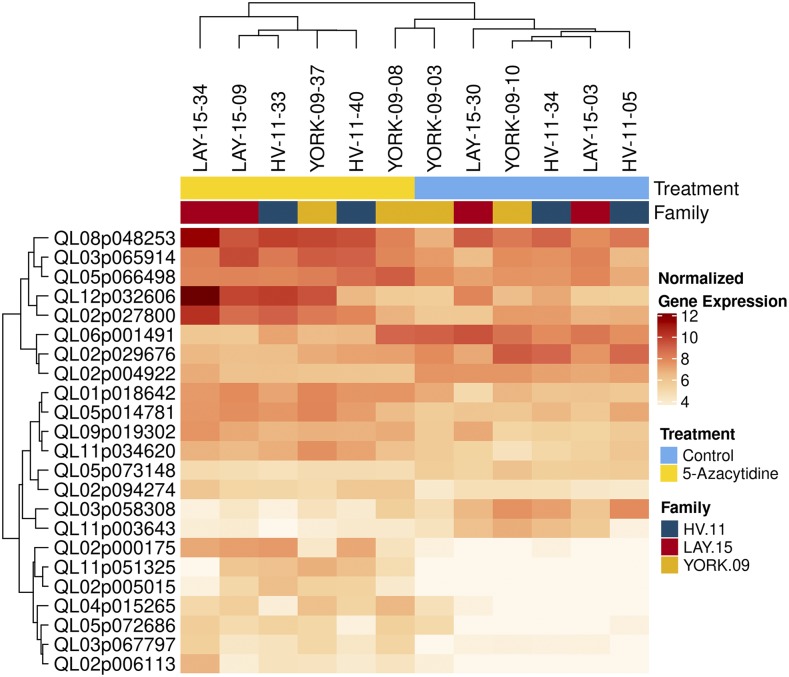
Heatmap of gene expression for the genes that were differentially expressed between *Quercus lobata* seedlings treated with demethylating agent 5-Azacytidine (n = 6) *vs.* control (n = 6). Cells show the normalized gene expression for a given gene and individual (darker colors are more highly expressed). Both samples and genes are clustered by similarity in gene expression (using the “complete” method of the hclust function in R).

We collected leaf tissue at the end of the experiment from 12 seedlings (n = 6 treatment, n = 6 control) for RNA-seq analysis. We found that a total of 23 genes were differentially expressed in demethylated seedlings, 17 of which were upregulated and 6 downregulated (Figure 4). A PCA of genes differentially expressed under demethylation treatment shows that samples cluster primarily by treatment (Figure S5a) while a PCA of gene expression across all genes shows samples loosely clustering by family (Figure S5b).

Families responded to the demethylation treatment differently. When families were analyzed separately, 93 genes were differentially expressed in the demethylation treatment for HV seedlings (44 up, 49 down), 140 genes in LAY (80 up, 60 down), and 135 genes in YORK (63 up, 72 down). Few of these genes were upregulated or downregulated in more than one family (nine were upregulated in two families, and four were downregulated in two families).

Gene ontology enrichment analysis identified terms that were overrepresented in genes that were upregulated and downregulated in seedlings treated with 5-Azacytidine (Table S3), although many of the overrepresented terms were based on only one or two DE genes due to the low number of annotated DE genes and thus caution is warranted in their interpretation. Upregulated genes were enriched for eight terms, including “transferase activity, transferring acyl groups” and “plant-type secondary cell wall biogenesis”. Downregulated genes were enriched for ten terms, including “ATP synthesis coupled proton transport,” “protein transport,” and “membrane.” We also observed enrichment of GO terms for DE genes within 2/3 sampled families, including terms involved in “lignin catabolic process”, “translation”, “jasmonic acid biosynthetic process”, “RNA 3`-end processing”, and “RNA polyadenylation” (Table S3).

### Effects of 5-Azacytidine on soil microbiome

We did not find any significant differences in the alpha diversity or community composition of the soil microbiome for samples treated with 5-Azacytidine *vs.* controls for either 16S (bacteria and archaea) or ITS (fungi) primers (*P* > 0.05 for all tests, Figure S6, Figure S7, Figure S8), but we caution this result might be due to limited sample size. For 16S, but not ITS, our sample sizes were sufficient to detect significant differences in community composition among seedling localities for the Bray-Curtis (*P* = 0.007), Jaccard (*P* = 0.003), and Chao estimators (*P* = 0.022, Figure S7).

## Discussion

Overall, demethylation of DNA, induced by the foliar application of 5-Azacytidine, was associated with several changes in growth and patterns of gene expression of 5-month old *Q. lobata* seedlings. The seedlings treated with the 5-Azacytidine showed signs of reduced growth, suggesting that developmental processes may have been disrupted by demethylation. This disruption may have been caused by genes that were differentially expressed between treated and untreated seedlings, consistent with the notion that methylation is associated with gene regulation.

Overall, 5-Azayctidine was effective at reducing genome-wide methylation levels on ∼5 month old *Quercus lobata* seedlings, with a magnitude similar to previous studies. Puy *et al.* (2017) observed a 21% relative reduction in overall genome-wide DNA methylation, while in this study we observed a 19.4% (6.7–43.2% depending on sequence context) average relative reduction in DNA methylation. Similar to Griffin *et al.* (2016), we also found that CG methylation showed lower levels of relative reduction than CHH methylation following 5-Azacytidine treatment. While many previous studies have confirmed the demethylating effects of 5-Azacytidine (Burn *et al.* 1993; Fieldes *et al.* 2005; Griffin *et al.* 2016; Tatra *et al.* 2000), to our knowledge, ours is the first study to show that foliar application of 5-Azacytidine is effective at reducing genome-wide DNA methylation on seedlings > 2 months old, and seedlings of a woody species. Our results open up the possibility of using experimental demethylation in combination with temperature or drought treatments to improve our understanding of the contribution of DNA methylation to tree response to environmental change (Bräutigam *et al.* 2013). However, the broad-scale, genome-wide reduction in DNA methylation caused by 5-Azacytidine prevents a deeper mechanistic insight into how DNA methylation at specific genomic regions or contexts is linked to particular phenotypic or gene expression changes, which could be addressed through the continued development of targeted demethylation approaches that are designed to remove methylation at specific regions of the genome (Gallego-Bartolomé *et al.* 2018).

The association of 5-Azacytidine treatment with reduced growth has been observed in studies with the application during seed germination, potentially due to a disruption of root development (Bossdorf *et al.* 2010; Kanchanaketu and Hongtrakul 2015; Pecinka and Liu 2014; Puy *et al.* 2017). However, with foliar application of 5-Azacytidine, Puy *et al.* (2017) did not observe a reduction in growth for *Taraxacum brevicorniculatum*, which is in contrast to our study where we observed a 50% reduction in new growth. Whether the reduced growth that we observed is due to species-specific responses to foliar application of 5-Azacytidine, or to the way we stimulated new growth by pruning seedlings, is unknown and more studies are needed to assess the growth consequences of foliar application of 5-Azacytidine across species.

Fluctuating asymmetry in leaves is one potential effect of 5-Azacytidine treatment because deviations from bilateral symmetry are commonly associated with developmental perturbations and plant stress (Palmer and Strobeck 1986; Viscosi 2015) and DNA methylation has been a proposed as a potential factor in leaf morphology (Herrera and Bazaga 2013). However, differences in fluctuating asymmetry associated with treatment by 5-Azacytidine were not statistically significant and were weaker than differences in fluctuating asymmetry across families, suggesting the natural amount of variation in fluctuating asymmetry across families and populations may swamp potential effects caused by 5-Azacytidine.

Consistent with some previous studies, we found a slight trend toward upregulation of genes following treatment with 5-Azacytidine, with 17 of the 23 differentially expressed genes across showing higher expression levels in the treatment group. In plants, DNA methylation in the promoter region of genes can inhibit transcription and demethylation can lead to the reactivation of silenced genes (Baubec *et al.* 2014; Burn *et al.* 1993; Griffin *et al.* 2016). Griffin *et al.* (2016) found that upregulated genes following application of 5-Azacytidine were enriched for transposable elements, which is consistent with the observation that DNA methylation is commonly associated with the silencing of transposons (Law and Jacobsen 2010). However, a non-trivial number of genes are often found to be down-regulated following DNA demethylation, which highlights the varied effects of DNA methylation on transcriptional regulation (Griffin *et al.* 2016; Harris *et al.* 2018). Because we did not obtain whole methylome sequences for all the seedlings, we are unable to address whether the demethylated regions overlapped with transposons. A potential explanation for the growth differences we observed in treated seedlings is that the expression and mobilization of transposable elements following demethylation shaped differences in the amount of new growth. This mechanism has been proposed by others (Bossdorf *et al.* 2010; Cheng *et al.* 2004; Johannes *et al.* 2009; Kanchanaketu and Hongtrakul 2015) and deserves further study

In our study, low amount of differentially expressed genes between demethylated and control seedlings was surprising given the large observed differences in growth. The low amount of differentially expressed genes may be due in part to the demethylation treatment not being severe enough to cause detectable changes in gene expression, or because population- or family-specific gene expression responses in valley oak would reduce the limit the number of genes differentially regulated across all samples (Mead *et al.* 2019). Additionally, a mismatch between the timing of sampling for gene expression (*e.g.*, at the end of the experiment) and gene expression impacts on phenotypic development, which could occur early in leaf and stem formation, would reduce the power to detect differentially expressed genes that impact growth. Obtaining samples across the duration of the study in addition to increasing the overall number of samples would likely permit a more comprehensive and powerful analysis of the functional roles of genes responding to 5-Azacytidine treatment.

## Conclusions

Resolving the connections between DNA methylation, phenotypic variation, growth, and gene expression in plants is crucial to understanding the role of epigenetic modifications in natural selection and plant response to rapid environmental change. We found that application of 5-Azacytidine led to overall genome-wide reductions in DNA methylation across all sequence contexts and was associated with phenotypic changes and differential gene expression in an ecologically important, non-model woody species. We also confirmed that these changes did not seem to be associated with changes in the soil microbiome, though increased sampling in future studies may reveal a role for the soil microbiome in mediating phenotypic responses to demethylation. Though limited in terms of sample size and breadth of phenotypic measurements, our findings provide support for the involvement of DNA methylation in shaping variation in phenotype and gene expression. Further studies, potentially with targeted demethylation approaches in combination with drought and temperature treatments, are needed to further build the mechanistic links between DNA methylation, phenotype, and gene expression to resolve the role of DNA methylation in plant adaptation to the environment and response to environmental change.
